# From Plains to Mountains: Results of Current and Future Climatic Suitability Analysis for *Crocus sativus* L. Cultivation in Italy

**DOI:** 10.3390/plants15050693

**Published:** 2026-02-25

**Authors:** Luca Giupponi, Davide Pedrali, Annamaria Giorgi

**Affiliations:** 1 Centre of Applied Studies for the Sustainable Management and Protection of Mountain Areas (CRC Ge.S.Di.Mont.), University of Milan, 25048 Edolo, Italy; luca.giupponi@unimi.it (L.G.); anna.giorgi@unimi.it (A.G.); 2 Department of Agricultural and Environmental Sciences—Production, Landscape and Agroenergy (DiSAA), University of Milan, 20122 Milan, Italy

**Keywords:** *Crocus sativus*, saffron, MaxEnt, mountain areas, climate change, suitable habitat, species distribution models

## Abstract

This research assessed current and future climatic suitability for *Crocus sativus* L. cultivation across Italy, using species distribution models. A dataset of 721 georeferenced points from sites consistently producing top-quality saffron was combined with bioclimatic variables from the CHELSA v2.1 database. Habitat suitability was modelled with MaxEnt and projected under current (2025) climatic conditions and future scenarios for mid-century (2055) and late-century (2085), based on the GFDL-ESM4 model and the SSP3-7.0 emission scenario. The MaxEnt model showed moderate predictive performance (AUC = 0.73 ± 0.02; TSS = 0.37 ± 0.03), which is consistent with the broad ecological tolerance of *C. sativus*. Current suitable areas (90,049 km^2^) are mainly in central and northern Italy, especially along the hilly Apennines and much of the Po Plain. Response curves indicate that optimal saffron cultivation occurs mainly under moderately continental conditions, with moderate to high temperature seasonality (6.5–7.5 °C), cool winter temperatures (mean of the driest quarter 0–3.5 °C), and relatively high precipitation during the wettest month (150–250 mm). Future projections show an expansion of suitable areas (124,552 km^2^ in 2055; 123,868 km^2^ in 2085) and a spatial shift from lowlands and coasts toward hilly and mountain regions of the Apennines, the Alps, and the main islands. These findings can support farmers, land managers, and policy-makers in informed planning and sustainable management of saffron cultivation under climate change.

## 1. Introduction

*Crocus sativus* L., commonly known as saffron crocus, is a perennial (geophyte) male-sterile triploid species (2n = 3× = 24) of the Iridaceae family cherished for its stigmas which are used to produce saffron, the world’s most valuable spice [1]. The saffron crocus is native to Greece, specifically to the Attica region [2,3], and its cultivation dates back more than 3000 years [4]. This species has been cultivated throughout the Mediterranean basin, including ancient Greece and Persia, but also outside of this area, such as in Iran and India [5] because its distinctive flavour, aroma, and colour have led it to occupy a significant place in culinary traditions, medicinal practices, and cultural rituals worldwide, encompassing many cultures, continents, and civilizations.

Currently, the main saffron-producing countries are Iran (>150,000 kg per year) and India (>15,000 kg per year) [6], but *C. sativus* is also cultivated in some European countries (mainly Greece, Spain, Portugal, France and Italy) whose spice productions are significantly lower compared to those of the two aforementioned Asian countries.

In Italy, saffron has been cultivated since Roman times [5] and today this country is one of the main producers among those of the Mediterranean basin (450–600 kg of spice per year). In Italy, *C. sativus* is cultivated by hundreds of small farms scattered throughout the national territory, from the southern regions and islands [7,8] to the Alps [9,10,11] but mainly in hilly and sub-mountain areas [12]. Recent studies have shown that the majority (>80%) of the saffron produced by Italian farms is of excellent quality [13,14] according to the standards defined by ISO 3632 which classifies the spice based on flavour strength, aroma strength, colouring strength and moisture content. Furthermore, Italian saffron is sold at a medium-high price (around 23.7 euros per gram) and is produced using sustainable and low-input agricultural techniques: only 1% of Italian farmers use agrochemicals, less than 10% irrigate fields, and only 40% of the farms are mechanized [12].

The high quality, economic (and historical-cultural) value, environmental sustainability of the production chain, and the possibility of cultivation throughout much of the national territory, make saffron a strategically important agri-food and herbal resource for Italy and the European Union (EU). The production of saffron (as performed in Italy) can indeed contribute to achieving the goals of the European “Farm to Fork” strategy, including making the European food system more sustainable, primarily by reducing the use of pesticides in agriculture, increasing organic farming, and promoting crop diversification and biodiversity conservation [15]. Furthermore, the saffron supply chain can contribute to the sustainable development of Italy’s marginal and mountainous territories following the National Recovery and Resilience Plan (NRRP) launched by the Italian government to address the economic and social challenges arising from the COVID-19 pandemic and to promote economic recovery, innovation, and sustainability [16]. Within the framework of the NRRP, Italy has funded various research and development projects, including “Agritech—National Center for the Development of New Agricultural Technologies” (https://agritechcenter.it/it/), of which the activities of Spoke 7 (where this research falls) focus on the development of marginal areas by promoting multifunctional and sustainable production systems.

One of the still unaddressed topics of fundamental importance for initiating actions (or investments) to support the Italian saffron supply chain is the analysis of the climatic conditions most suitable for producing high-quality saffron and, above all, the identification of the geographic areas where such conditions are likely to occur in the coming decades under ongoing climate change [17]. Indeed, although *C. sativus* is a hardy species [8] with high genetic variability [18], its propagation occurring exclusively through vegetative/asexual means (corm duplication) [19] prevents the evolution of populations, hence their ability to adapt to rapid climate change.

This research aims to predict current and future areas climatically suitable for *C. sativus* cultivation in Italy using species distribution models (SDMs) [20,21]. Specifically, based on occurrence data of the saffron crocus (which allows the production of high-quality spice) across the Italian territory and on the climatic characteristics of the areas where it is cultivated, maps of climatically suitable areas for the cultivation of this plant in Italy will be produced, thereby providing useful information for its in situ (on farm) conservation and cultivation, as well as for the management and valorization of this agri-food and herbal resource and its related supply chains.

## 2. Materials and Methods

### 2.1. Occurrence Data Source

The geographical coordinates of 721 areas (occurrence points) where Italian top-quality saffron is produced were collected from the database of the “Val.Te.Mo.” association (https://www.valtemo.it accessed on 10 November 2025). This association, which aims to enhance the resources of mountain territories of Italy, has been conducting qualitative analyses (according to the ISO 3632 1,2:2010-2011 standard) of saffron produced in Italy in collaboration with the University of Milan. The “Val.Te.Mo.” database, covering the period 2015–2022, represents the most comprehensive and up-to-date dataset of saffron production in Italy, including both professional farmers and hobbyist producers, and was accessed following a formal agreement.

**Figure 1:**
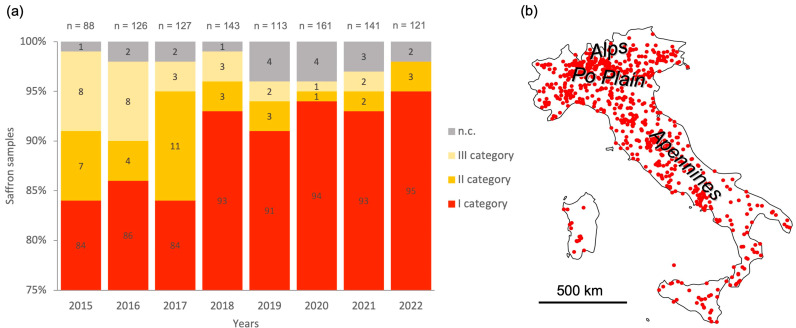
Number and quality of saffron samples analyzed in Italy from a qualitative point of view between 2015 and 2022 (**a**), and sites (red dots) where top-quality (first-category) saffron was produced (**b**). Quality categories follow the ISO 3632 standard: category I, first-quality saffron; category II, second-quality saffron; category III, third-quality saffron; n.c., saffron not classifiable according to ISO 3632. Data sources: 2015–2018, [13]; 2019–2022, “Val.Te.Mo.” Association database (original data).

Figure 1 shows the quality and the number of saffron samples analyzed from 2015 to 2022, as well as the sites where top-quality saffron (first-category according to ISO 3632 standards) has been produced. For this study, only the geographical coordinates of areas producing top-quality saffron for more than one year were included (721 occurrences). According to ISO 3632, top-quality saffron is characterized by: picrocrocin ≥70 (flavour strength), safranal 20–50 (aroma strength), crocin ≥200 (colouring strength) and moisture content ≤12%.

The coordinates of the 721 occurrence points were arranged in a table, which was then imported into the R 4.3.2 software [22] to carry out statistical analysis aimed at identifying climatically suitable areas for *C. sativus* cultivation.

### 2.2. Environmental Data

Nineteen bioclimatic variables were obtained from the CHELSA v2.1 database [23] at a spatial resolution of 30 arc-seconds (~1 km). These variables represent climatic conditions for the current reference period (corresponding to the reference period 2011–2040, i.e., centred around 2025) and capture annual trends, seasonality, and extreme or limiting environmental factors (Table S1).

To reduce multicollinearity among predictors, a variance inflation factor (VIF) analysis was performed using the “vifstep” function from the “usdm” R package [24]. Variables with VIF > 5 were iteratively removed and the remaining variables were used for modeling.

### 2.3. Species Distribution Modelling and Model Evaluation

Species distribution modelling was performed using the Maximum Entropy algorithm (MaxEnt) [25,26,27] as implemented in the dismo R package [28].

A total of 10,000 background points were randomly generated within Italy to characterize the environmental conditions available to saffron cultivation. Model calibration used a random partition of the occurrence data, with 75% of records for training and 25% for testing. To assess model stability and reduce sampling bias, a five-fold cross-validation was applied.

The relative contribution of each bioclimatic variable was extracted from the MaxEnt model, and response curves were generated to examine the influence of environmental predictors.

Model performance was evaluated using the area under the curve (AUC) of the receiver operating characteristic (ROC), a widely used metric for presence-only data [25,29]. In addition, the true skill statistic (TSS) was calculated as a threshold-dependent measure of model accuracy. Metrics were computed separately for each cross-validation fold, and mean values and standard deviations were used to assess variability among folds.

### 2.4. Current Climate Suitability Analysis

The calibrated MaxEnt model was used to predict current habitat suitability for saffron cultivation across Italy. Model outputs were expressed as continuous suitability values ranging from 0 (low) to 1 (high).

To create a binary suitability map, a threshold was applied to the continuous predictions, selected based on the maximum sensitivity plus specificity criterion, which maximizes the sum of sensitivity (true positive rate) and specificity (true negative rate), ensuring a balanced trade-off between omission and commission errors [30]. Areas exceeding the threshold were classified as climatically suitable for saffron cultivation.

### 2.5. Future Climate Suitability Analysis

Future climate projections were generated for mid-century and late-century periods, corresponding to the 30-year time windows 2041–2070 and 2071–2100, respectively, and hereafter referred to by their central years (2055 and 2085). Bioclimatic variables for these periods were obtained from the CHELSA v2.1 database [23], downscaled to the same spatial resolution as current data.

The GFDL-ESM4 global climate model was used to simulate future climate conditions. GFDL-ESM4 is a fully coupled atmosphere-ocean general circulation model of the latest generation [31], designed to provide projections under different shared socio-economic pathways (SSPs).

The SSP3-7.0 scenario (“Regional Rivalry—high challenges to mitigation and adaptation”) was selected as it represents a plausible pathway with high greenhouse gas emissions and limited global mitigation efforts, providing a conservative estimate of potential impacts of climate change on saffron cultivation [32,33,34].

To evaluate areas of environmental novelty and potential extrapolation under future conditions, multivariate environmental similarity surface (MESS) analyses were conducted in R environment. MESS maps identify locations where one or more climatic variables fall outside the range observed under current conditions (MESS < 0), highlighting regions where predictions should be interpreted with caution [35].

Future habitat suitability maps for 2055 and 2085 were produced as both continuous and binary maps, using the same threshold applied for the current distribution. Areas with MESS < 0 were excluded from the binary maps to avoid unreliable predictions in regions with novel climatic conditions.

## 3. Results

### 3.1. Areas of Current Climate Suitability

**Figure 2:**
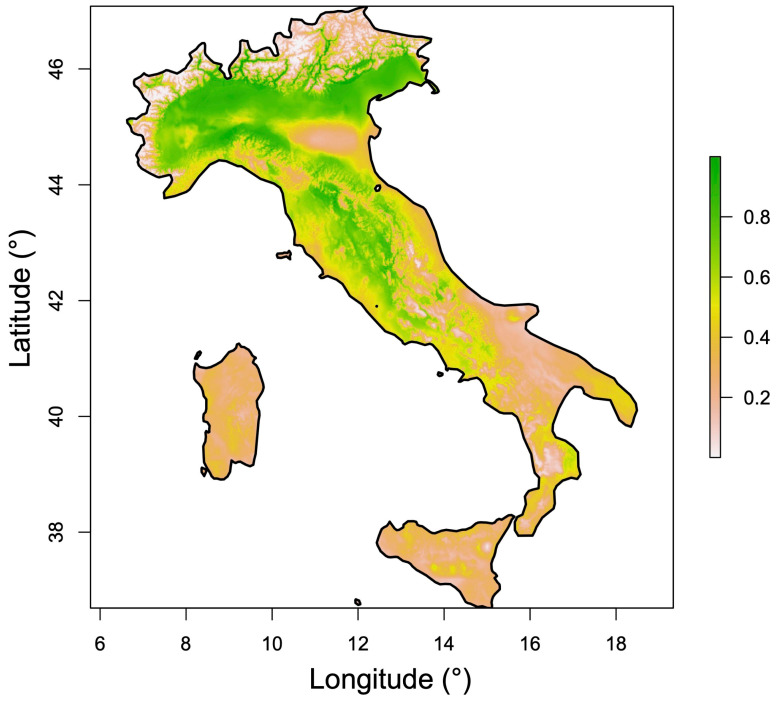
Current climatically suitable areas for *C. sativus* cultivation in Italy, expressed as continuous habitat suitability.

The MaxEnt model predicted the current climatically suitable habitat for *C. sativus* across Italy (Figure 2). Suitable areas are mainly concentrated in central and northern Italy, particularly along the hilly Apennines (Emilia-Romagna, Tuscany, Umbria, Lazio, and Abruzzo regions) and large parts of the Po Plain. Lower suitability was predicted in southern Italy and on the major islands (Sicily and Sardinia).

**Figure 3:**
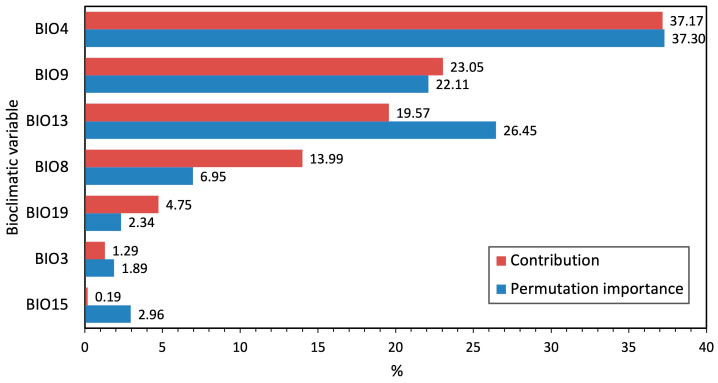
Importance of bioclimatic predictors in the species distribution model, expressed as percent contribution and permutation importance. Variable codes correspond to those reported in Table 1 and Table S1.

Table 1 shows the seven bioclimatic variables retained after multicollinearity analysis (those with VIF < 5), while Figure 3 illustrates the relative contribution of each predictor, highlighting the importance of temperature seasonality (BIO4), mean temperature of the driest quarter (BIO9), precipitation of the wettest month (BIO13), and mean temperature of the wettest quarter (BIO8). The response curves of these variables (Figure S1) indicate that optimal saffron cultivation is associated with moderate to high temperature seasonality (6.5–7.5 °C), typical of moderate continental climatic conditions, a mean temperature of the driest quarter (winter months) ranging from 0 °C to 3.5 °C, precipitation of the wettest month between 150 mm and 250 mm, and a mean temperature of the wettest quarter exceeding 10 °C.

**Figure 4:**
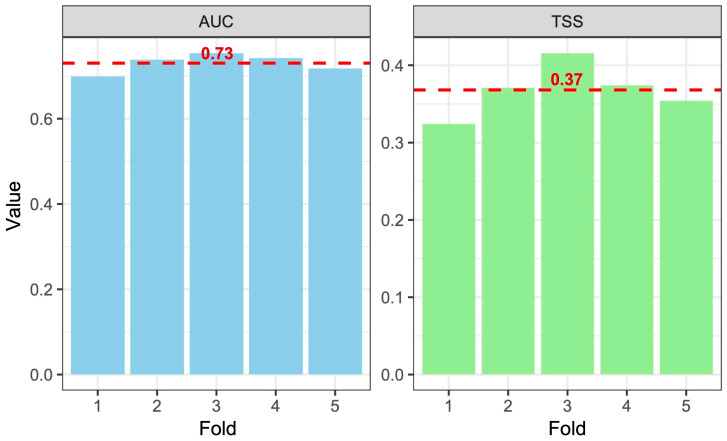
Model evaluation results based on five-fold cross-validation. Bar plots show AUC and TSS values for individual folds, with the dashed red line indicating the mean value across folds.

Model evaluation based on five-fold cross-validation indicated a moderate predictive performance of the MaxEnt model (Figure 4). The mean AUC (0.73 ± 0.02) and TSS (0.37 ± 0.03) values indicate an acceptable discrimination capacity, supporting the use of the model to identify areas potentially suitable for saffron cultivation.

**Figure 5:**
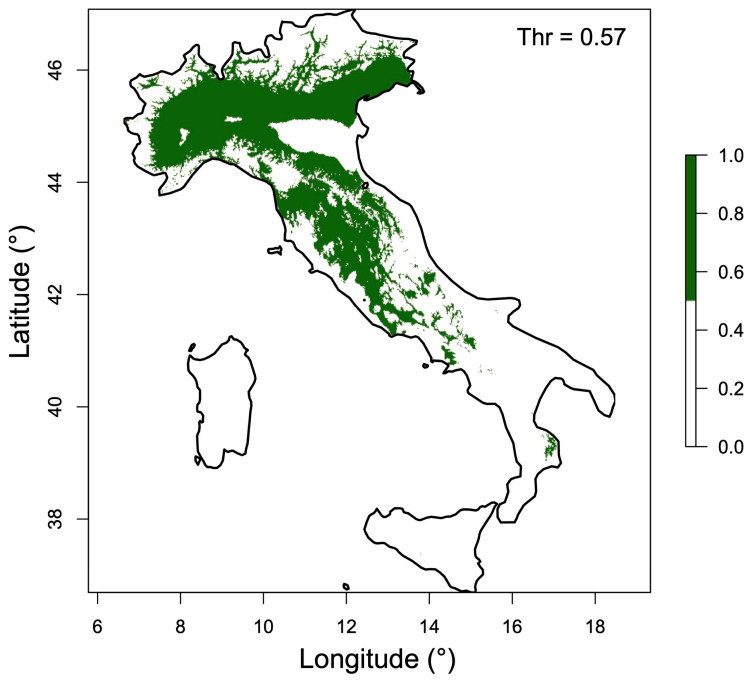
Binary map of current habitat suitability for *C. sativus* in Italy. Areas classified as suitable are shown in green, based on the selected threshold (Thr) applied to the continuous suitability prediction.

By applying a threshold of 0.57, derived from the maximum sensitivity plus specificity criterion, a binary map distinguishing suitable from unsuitable areas was generated (Figure 5). Areas classified as suitable covered approximately 90,049 km^2^ and largely corresponded to regions with a long history of saffron cultivation as well as sites producing high-quality saffron, supporting the consistency between occurrence records and the predicted climatic suitability (Figure 1).

### 3.2. Areas of Future Climate Suitability

**Figure 6:**
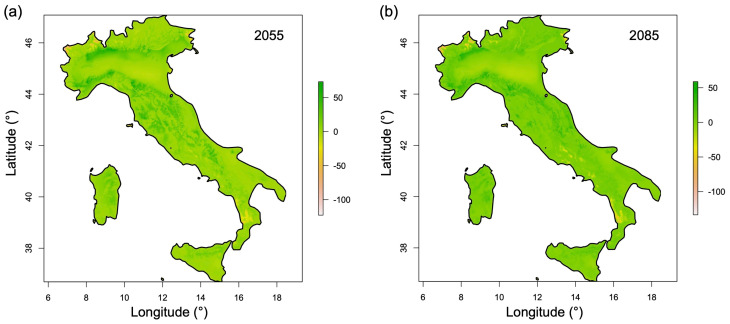
MESS maps for Italy showing the multivariate environmental similarity for *C. sativus* under future climate scenarios: 2055 (**a**); 2085 (**b**). Positive values indicate areas with environmental conditions similar to the present, while negative values highlight areas with novel conditions not represented in the current climate.

Figure 6 shows the MESS maps for Italy under future climate scenarios for 2055 and 2085. In both time horizons, areas characterized by negative MESS values (indicating novel climatic conditions) are very limited in extent and spatially fragmented. Most of the Italian territory displays positive MESS values, suggesting that future climatic conditions largely remain within the range of environmental conditions observed under the current climate. The restricted spatial extent of areas with MESS < 0 indicates that future habitat suitability projections for *C. sativus* are mostly based on interpolation rather than extrapolation, and therefore can be considered reliable for the majority of the study area.

**Figure 7:**
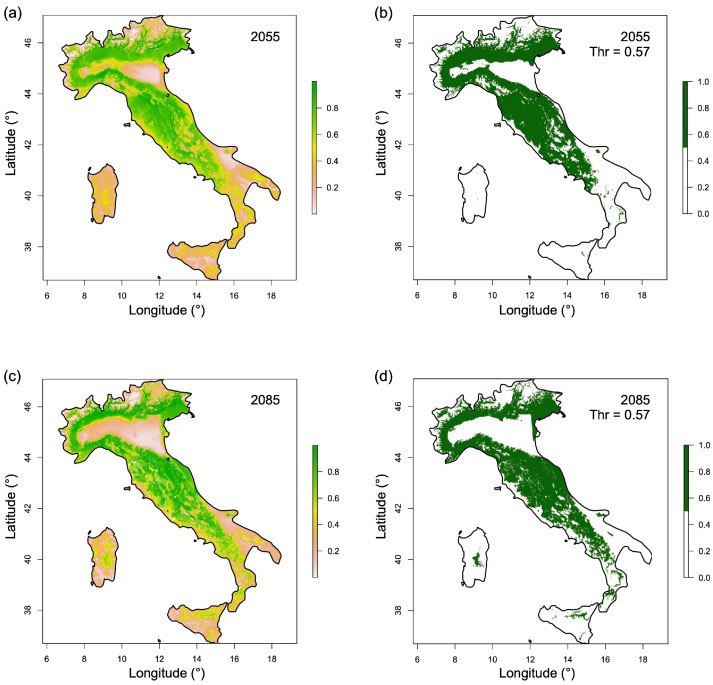
Predicted habitat suitability for *C. sativus* in Italy under future climate scenarios. Continuous probability maps for 2055 (**a**) and 2085 (**c**) and corresponding binary maps based on the threshold (Thr) for 2055 (**b**) and 2085 (**d**). Areas classified as suitable in the binary maps are shown in green. The threshold used to generate binary maps is the same as for the current distribution (Figure 5).

After excluding areas with MESS < 0, continuous habitat suitability maps for 2055 and 2085 (Figure 7a,c) reveal a marked spatial reorganization of climatically suitable areas for *C. sativus* cultivation. Suitable conditions progressively decline in lowland areas, particularly in the Po Plain, where they are almost completely absent by 2085.

Analysis of the binary suitability maps (Figure 7b,d) indicates that the total extent of suitable areas is greater under future scenarios than under current conditions, reaching 124,552 km^2^ in 2055 and 123,868 km^2^ in 2085. However, suitable habitats increasingly concentrate in hilly and mountainous regions of the Italian peninsula (the Alps and the Apennines), as well as in mountain areas of the main islands (Sardinia and Sicily). By 2085, large portions of Italy’s coastal areas are projected to no longer present climatically suitable conditions for *C. sativus* cultivation, with the exception of coastal zones in Friuli Venezia Giulia, Veneto, Liguria, Marche, and Abruzzo regions (Figure 7d).

## 4. Discussion

According to the results obtained from the MaxEnt species distribution model, current climatically suitable areas for *C. sativus* are mainly distributed across central and northern Italy, with a strong association with the Apennine range and parts of the Po Plain (Figure 2 and Figure 3). The total extent of suitable areas under current climatic conditions was estimated at approximately 90,049 km^2^, largely overlapping areas with a long tradition of saffron cultivation and areas known for high-quality production. This spatial coherence between occurrence records (Figure 1) and predicted suitability supports the reliability of the input dataset and the overall robustness of the modelling approach.

Model performance metrics (AUC and TSS) indicated a moderate but acceptable predictive accuracy (Figure 4). While these values are lower than those typically reported for narrowly distributed wild species [36], they are consistent with expectations for a cultivated, euriecious species such as *C. sativus*. Saffron is able to persist under a relatively wide range of climatic and pedological conditions [37]. Moreover, the occurrence data included both traditional and marginal cultivation sites, increasing environmental heterogeneity and consequently reducing model discrimination power. Similar patterns have been reported in SDM studies focused on crops or semi-domesticated species [38], where moderate AUC and/or TSS values still provide meaningful insights into large-scale climatic suitability.

The most influential bioclimatic predictors identified by the model were temperature seasonality (BIO4), mean temperature of the driest quarter (BIO9), precipitation of the wettest month (BIO13), and mean temperature of the wettest quarter (BIO8) (Figure 3). Temperature seasonality (BIO4), which reflects annual temperature variability, emerged as the most important predictor, highlighting the role of adequate thermal amplitude in supporting the phenological cycle of saffron. *Crocus sativus* does not require cold winters to break corm dormancy, as dormancy occurs during summer rather than winter. Floral induction takes place during summer dormancy under warm conditions, while non-optimal or overly stable thermal regimes may negatively affect flowering and yield. The importance of BIO9 and BIO8 indicates that temperatures during critical growth periods (corresponding to the driest and wettest quarters) are essential to limit physiological stress, support floral induction, and favor vegetative growth, provided that moisture levels are not excessively high. Precipitation of the wettest month (BIO13) further highlights the species’ sensitivity to excess rainfall, particularly during flowering, when prolonged wet conditions can negatively affect yield and quality [7,39,40]. The response curves (Figure S1) confirm that optimal suitability is associated with temperate climates characterized by intermediate precipitation levels and marked, but not extreme, seasonal temperature fluctuations. *C. sativus* tolerates winter temperatures down to −15 to −20 °C and requires summer temperatures of 23–27 °C for optimal flowering, with blooms triggered by autumn temperatures of 15–17 (20) °C [41,42,43]. The combination of these climatic constraints explains the concentration of suitable areas in the Apennine and Alpine regions, particularly at elevations where continental and temperate climatic conditions coexist.

Future projections (2055 and 2085) indicate that climatically suitable areas for the cultivation of *C. sativus* will largely remain within the environmental space observed under current conditions, as highlighted by the limited extent of regions with MESS < 0 (Figure 6). This confirms that the majority of future habitat suitability predictions are based on interpolation and can therefore be considered reliable. Notably, suitable habitats are projected to decline in lowland areas, particularly in the Po Plain, the most important agricultural region in Italy, which is expected to lose its climatic suitability for saffron cultivation by 2085. Conversely, hilly and mountainous areas of the Apennines, the Alps, and the main islands (Sardinia and Sicily) are expected to retain or even expand their suitability, resulting in a spatial shift in potential cultivation zones. Binary maps indicate an overall increase in suitable area (124,552 km^2^ in 2055; 123,868 km^2^ in 2085) relative to the current extent (90,049 km^2^), reflecting this redistribution from plains to higher elevations (Figure 7), with suitable conditions projected on the Alps even above 1500 m a.s.l. and on the Apennines above 1200 m a.s.l.

Although this study assessed the potential current and future distribution of *C. sativus* in Italy by considering only climatic variables, which represent the primary environmental drivers influencing its growth and cultivation [44], other environmental factors (e.g., slope, land use, and infrastructure) should be taken into account to identify areas where saffron could be effectively cultivated. In fact, several mountainous regions of Italy are characterized by steep slopes that prevent the cultivation of *C. sativus* (and other crops) unless costly hydraulic-agrarian interventions, such as terracing, are implemented. Similarly, some mountainous areas are unsuitable for agricultural activities due to limited accessibility and the absence of adequate road infrastructure. In addition, some soils within the climatically suitable mountainous areas highlighted in Figure 7 may present chemical or physical properties that are unfavorable for saffron cultivation. Although *C. sativus* is a relatively hardy species, it performs best in well-drained soils with neutral to slightly basic pH, conditions that are essential for achieving high saffron yields [8,37,45].

The analysis of land use in the mountainous areas of Italy is also of fundamental importance for identifying territories (and fields) where it will be possible to cultivate *C. sativus* and produce saffron. If current land use patterns remain unchanged over the coming decades, fewer fields will be available for saffron cultivation. Indeed, today a large portion of the mountainous areas of the Alps and the Apennines is covered by forests (while the plains are largely devoted to agriculture), which represent habitats unsuitable for saffron cultivation: more than 60% of Italy’s forested area is located in mountain municipalities [46], and this area has increased by over 570,000 hectares in the last 30 years [37].

The increase in forest cover in the mountainous areas of Italy, at the expense of meadows, pastures, and arable fields, is primarily driven by socio-economic factors that have been the main cause of land abandonment in these territories for over half a century [47]. In Italy, various instruments have been implemented in recent decades to counteract the abandonment of mountain and marginal areas, and currently, several NRRP funds have been committed to projects aimed at enhancing the valuable agri-food resources of these territories (such as saffron) through the establishment or strengthening of traditional and/or innovative supply chains that can promote the sustainable development of these areas.

The results of this research could be highly useful for land managers and policymakers, enabling them to identify, promptly, the mountainous areas in Italy where saffron production may be feasible in the coming decades and to initiate targeted policies and actions accordingly. This study thus exemplifies how researchers can provide tools to support initiatives aimed at safeguarding (agro-)biodiversity and promoting local agri-food excellence, of which Italian mountain areas are particularly rich [48]. We therefore suggest that SDMs be increasingly applied not only to wild species but also to crops of agricultural and food interest, in order to identify the territories where they can be optimally cultivated while considering the effects of climate change.

For this approach to be replicated for other crops and/or geographic areas, however, several important prerequisites must be met, upon which the accuracy of the results depends, the most critical being the availability of comprehensive occurrence datasets. In this study, a large dataset of georeferenced points (more than 700) was used, representing locations where high-quality saffron is cultivated and produced in Italy. However, similar studies have been conducted in other geographical areas using much smaller occurrence datasets (which were likely the most comprehensive data available to the authors at the time) and whose results should therefore be interpreted with caution. This is the case for the analysis by Kumar et al. (2022) [44], who used only 20 geographic points across five countries (Spain, Morocco, Italy, Iran, and India) to identify, with MaxEnt, new areas in India suitable for saffron cultivation.

The collection and sharing of data on species distribution is therefore a fundamental action for the development of predictive maps that are as reliable as possible. In recent years, various open-source databases have been developed from which georeferenced points (occurrence records) related to the presence of a particular animal or plant species in a specific territory can be obtained. One of these is the Global Biodiversity Information Facility (GBIF) database (https://www.gbif.org accessed on 10 November 2025), which is an international network and data infrastructure funded by the world’s governments and aimed at providing, anyone and anywhere, open access data about all types of life on Earth [49,50]. Although GBIF is the largest source of open scientific data on the planet’s biodiversity (2.6 billion occurrence records in 2024), there is still much work to be done to integrate missing data regarding the distribution of many species. Consider that by the end of 2024, GBIF reported only 445 occurrences of *C. sativus* across 32 countries, with 121 georeferenced points for Italy and just 3 for Iran, the world’s leading saffron producer.

In the future, it would be desirable for researchers worldwide to make a greater effort to enrich species occurrence databases (for both wild and agricultural species), using the citizen science approach, which can play an increasingly important role in biodiversity monitoring [51], provided that citizens have adequate experience/knowledge regarding species identification and methods for collecting their occurrence data [52]. The creation of a sufficiently comprehensive dataset of *C. sativus* occurrences worldwide could allow for the replication of this study in other countries and a better understanding of the environmental requirements of this species. In addition, the collection of data on the average annual yield (as well as quality) of saffron produced in each geographical area could allow for the identification of the most productive areas, whose geographic coordinates could be used to develop maps predicting “optimal/high productivity” (current and future) areas for *C. sativus.*

Finally, regarding the identification of areas that will present suitable climatic conditions for saffron cultivation in the future, it is necessary to consider and monitor the actual SSP scenario that humanity will face, as well as the mitigation measures that will be adopted in the coming years and their effectiveness. Depending on the real SSP, which could be more or less favorable than that considered in this study, and the development of increasingly reliable global climate models [53], it would be advisable to regularly update the maps of areas with suitable climatic conditions for the cultivation of *C. sativus* in Italy, as these could undergo more or less significant changes. Nevertheless, this study represents the first attempt to predict areas where suitable climatic conditions currently exist and will exist in the future for *C. sativus* cultivation in Italy, based on the use of the most comprehensive set of occurrence points available to date and employing reliable global climate models and modern species distribution modeling tools.

## 5. Conclusions

This research identified the areas of Italy where climatic conditions suitable for the cultivation of *C. sativus* currently persist, as well as those expected to remain suitable in the coming decades, through the use of a vast dataset of this species occurrence in Italy and the application of MaxEnt SDMs. Currently, approximately 90,049 km^2^, mainly in central and northern Italy, present favorable climatic conditions, corresponding closely to historical saffron cultivation sites along the hilly Apennines and in large parts of the Po Plain. Models predict a significant shift in suitable areas over the next decades, with many plains, coastal zones, and low hills losing suitability by 2085. In contrast, hilly and mountain regions of the Alps and the Apennines are projected to retain or even expand the areas with suitable climatic conditions for *C. sativus* cultivation. The results obtained in this research could be useful not only to farmers who currently cultivate or will cultivate *C. sativus* but also to land managers and politicians, who will be able to make decisions with greater awareness and scientific data on how and where to allocate investments aimed at promoting and enhancing the Italian saffron chain.

## Figures and Tables

**Table 1:** Bioclimatic variables retained for modelling the distribution of *C. sativus* in Italy following multicollinearity analysis, with corresponding variance inflation factor (VIF) values for each predictor.

Code	Bioclimatic Variable	VIF
BIO3	Isothermality	1.62
BIO8	Mean Temperature of Wettest Quarter	2.11
BIO15	Precipitation Seasonality	3.00
BIO9	Mean Temperature of Driest Quarter	3.41
BIO13	Precipitation of Wettest Month	3.60
BIO19	Precipitation of Coldest Quarter	4.42
BIO4	Temperature Seasonality	4.84

## Data Availability

The raw data supporting the conclusions of this manuscript will be made available by the corresponding author, without undue reservation, to any qualified researcher.

## Supplementary Materials

The following supporting information can be downloaded at: https://www.mdpi.com/article/10.3390/plants15050693/s1, Table S1: Bioclimatic variables used for modelling the distribution of *C. sativus* in Italy; Figure S1: Response curves of the bioclimatic variables selected after collinearity analysis (VIF) for the *C. sativus* distribution model in Italy. Variable codes are the same as those reported in Table 1 and Table S1.
