# Supplementary figures and images for: From Plains to Mountains: Results of Current and Future Climatic Suitability Analysis for Crocus sativus L. Cultivation in Italy
Source: Plants (Basel). 2026 Feb 25;15(5):693. doi: 10.3390/plants15050693 (PMC12986869; doi:10.3390/plants15050693)

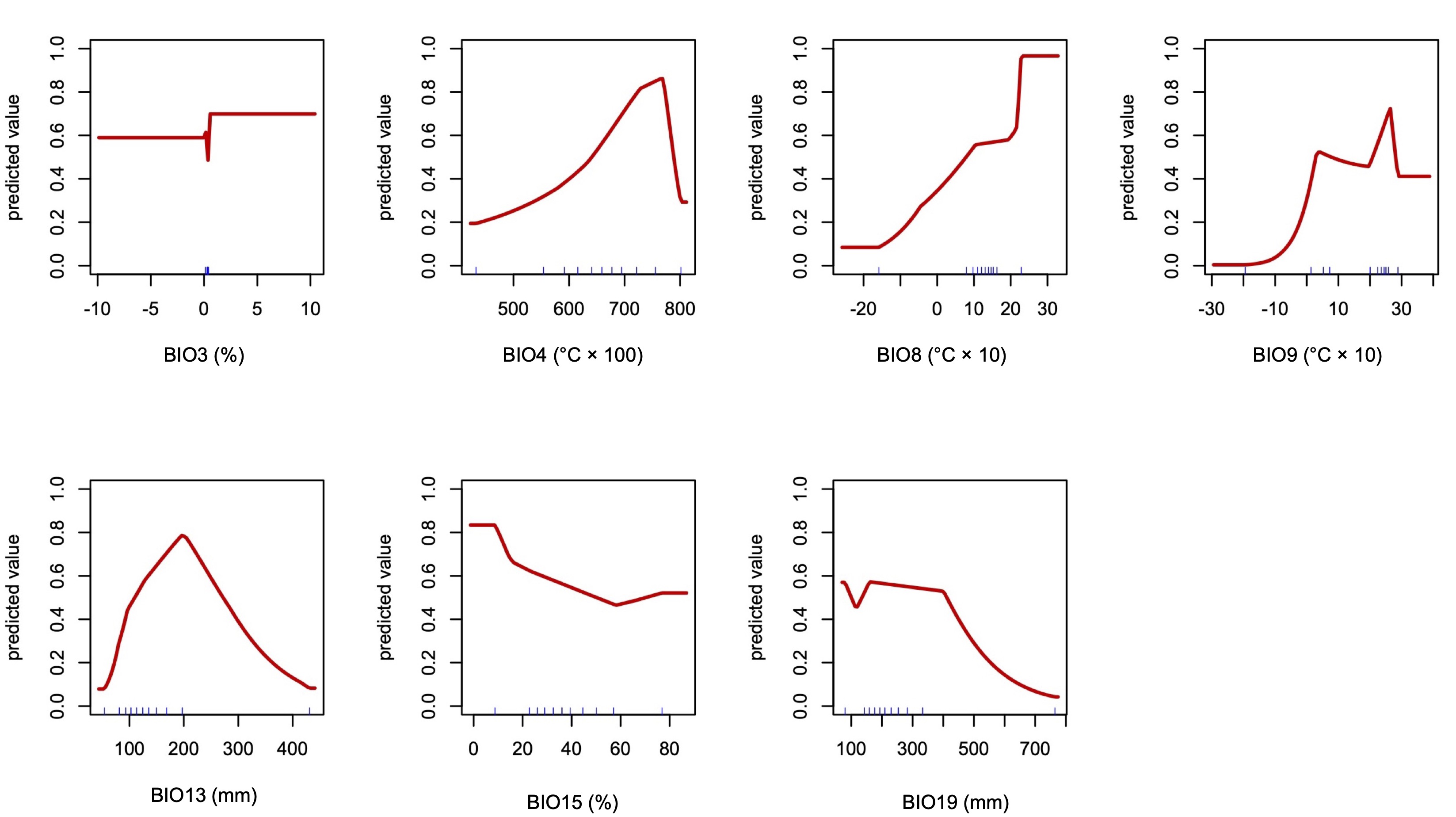

Supplement: Supplementary file 1 [file plants-15-00693-s001.zip › Supplementary Materials/Figure S1.jpg]
